# Supplementary material for: Low-input PacBio sequencing generates high-quality individual fly genomes and characterizes mutational processes
Source: Nat Commun. 2024 Jul 5;15:5644. doi: 10.1038/s41467-024-49992-6 (PMC11226609; doi:10.1038/s41467-024-49992-6)
Supplement: Supplementary file 1 — Supplementary Information [file 41467_2024_49992_MOESM1_ESM.docx]

**Supplementary Information for**

**Low-input PacBio sequencing generates high-quality individual fly genomes and characterizes mutational processes**

Hangxing Jia^1†*^, Shengjun Tan^1†*^, Yingao Cai^1,2†^, Yanyan Guo^1,2^, Jieyu Shen^1,2^, Yaqiong Zhang^1^, Huijing Ma^1^, Qingzhu Zhang^1,2^, Jinfeng Chen^2,3^, Gexia Qiao^1,2^, Jue Ruan^4*^, Yong E. Zhang^1,2*^

*^1^**Key Laboratory of Zoological Systematics and Evolution, Institute of Zoology, Chinese Academy of Sciences, Beijing, China.*

*^2^University of Chinese Academy of Sciences, Beijing, China.*

*^3^State Key Laboratory of Integrated Management of Pest Insects and Rodents, Institute of Zoology, Chinese Academy of Sciences, Beijing, China.*

*^4^**Shenzhen Branch, Guangdong Laboratory of Lingnan Modern Agriculture, Genome Analysis Laboratory of the Ministry of Agriculture and Rural Affairs, Agricultural Genomics Institute at Shenzhen, Chinese Academy of Agricultural Sciences, Shenzhen, China.*

^†^ Hangxing Jia, Shengjun Tan, and Yingao Cai contributed equally to this work.

^*^ Correspondence: [jiahangxing@163.com](mailto:jiahangxing@163.com); [tanshengjun@ioz.ac.cn](mailto:tanshengjun@ioz.ac.cn); [ruanjue@caas.cn](mailto:ruanjue@caas.cn); [zhangyong@ioz.ac.cn](mailto:zhangyong@ioz.ac.cn)

This document consists of Supplementary Figures and Supplementary References.

**Supplementary Figures**


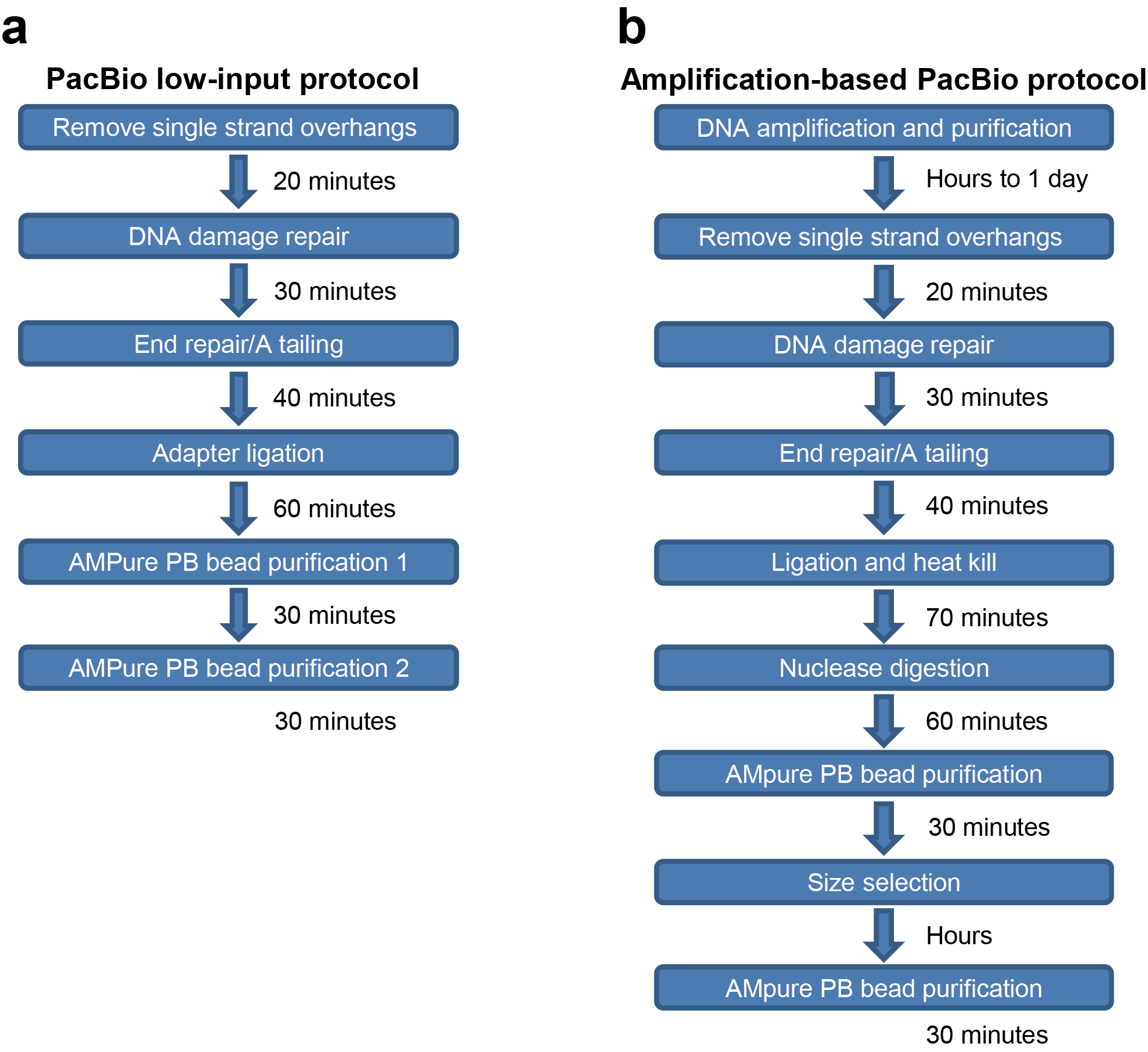


**Supplementary Fig. 1 | Prior PacBio low-input sequencing library preparation protocols.**

**a,** PacBio low DNA input protocol. This protocol has been implemented with SMRTbell Express Template Prep Kit 2.0. **b,** PacBio amplification-based ultralow DNA input protocol. While multiple protocols have been developed by PacBio and individual laboratories^1,2,3,4^, they all essentially involve DNA amplification as the initial step, followed by the preparation of standard PacBio libraries. The PacBio amplification-based ultralow DNA input protocol, being relatively more prevalent, is presented here. Abundant DNA generated by amplification is subjected to nuclease digestion to remove uncircularized DNA, with subsequent size selection for long DNA fragments. Additionally, heating is employed to inactivate the ligase, thus facilitating the digestion process.


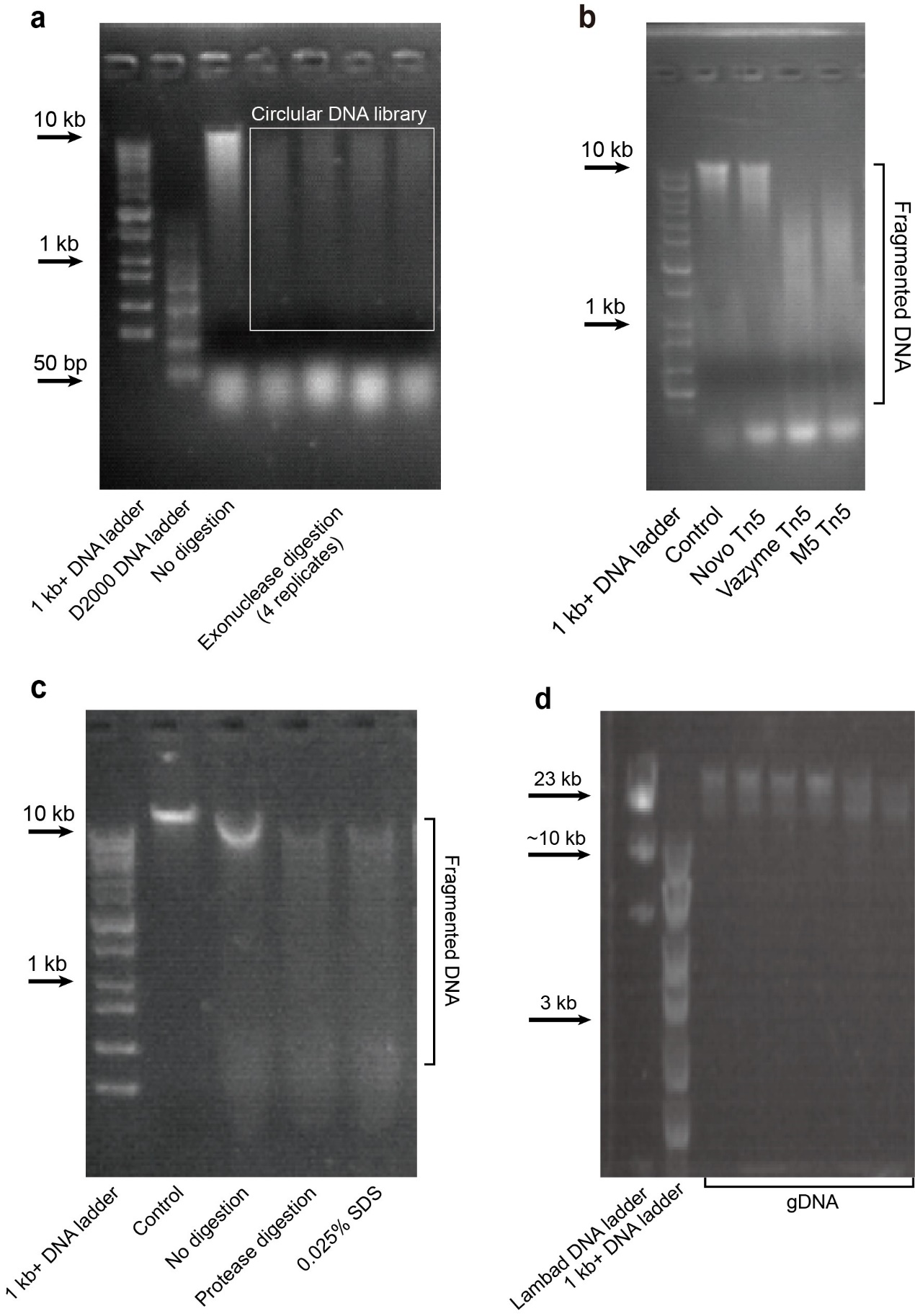


**Supplementary Fig. 2 | Experimental validations in the development of LILAP and quality analysis of single-fly genomic DNA.**

**a,** Reproducibility of LILAP. With the same DNA input, LILAP was run across five technical replicates. The first replicate (“No digestion”) served as a control without exonuclease digestion. For the remaining four replicates, noncircular DNA was digested by adding exonucleases. **b,** Fragmentation efficiency across Tn5 brands. Vazyme Tn5 and M5 Tn5 but not Novo Tn5 show a high fragmentation efficiency in the T4 DNA ligase buffer. **c,** Different conditions for the release of Tn5-tagged DNA. "Control" represents the condition without Tn5 fragmentation, while "No digestion" refers to the absence of protease digestion. These two experiments served as negative controls, while “Protease digestion” served as the positive control. Similar to proteases, SDS released DNA fragments, *i.e.*, fragmented DNA bands ranging between 1 kb and 10 kb. **d,** Genomic DNA (gDNA) analysis of individual flies by gel electrophoresis. Two lanes of length markers and six lanes of single-fly biological replicates are shown.


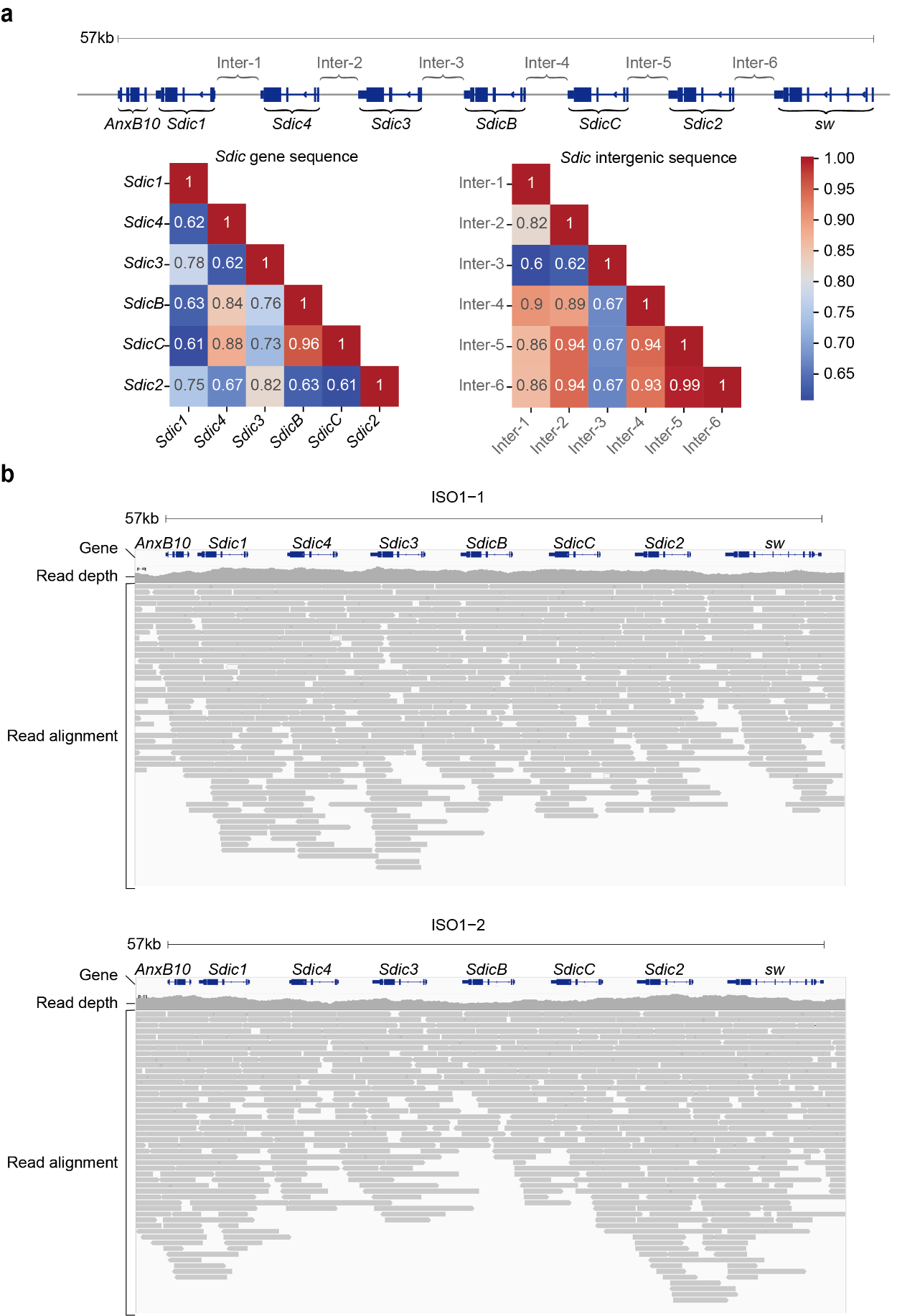


**Supplementary Fig. 3 | Analysis of the *Sdic* locus.**

**a**, Nucleotide sequence identity of various *Sdic* genes or the upstream intergenic loci. **b**, Distribution of sequencing depth and read alignment. It is noteworthy that a single contig (~6.7 Mb) in both ISO1-1 and -2 assemblies contains *Sdic* copies in the correct number and order.


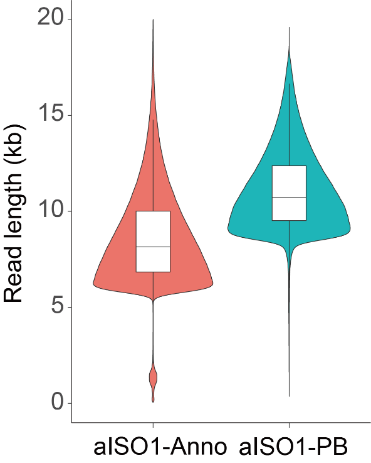


**Supplementary Fig. 4 | The length distribution of CCS reads for amplification-based PacBio sequencing data.**

The figure convention follows Fig. 2a (aISO1-Anno: *n* = 3158408; aISO1-PB: *n* = 2301518).


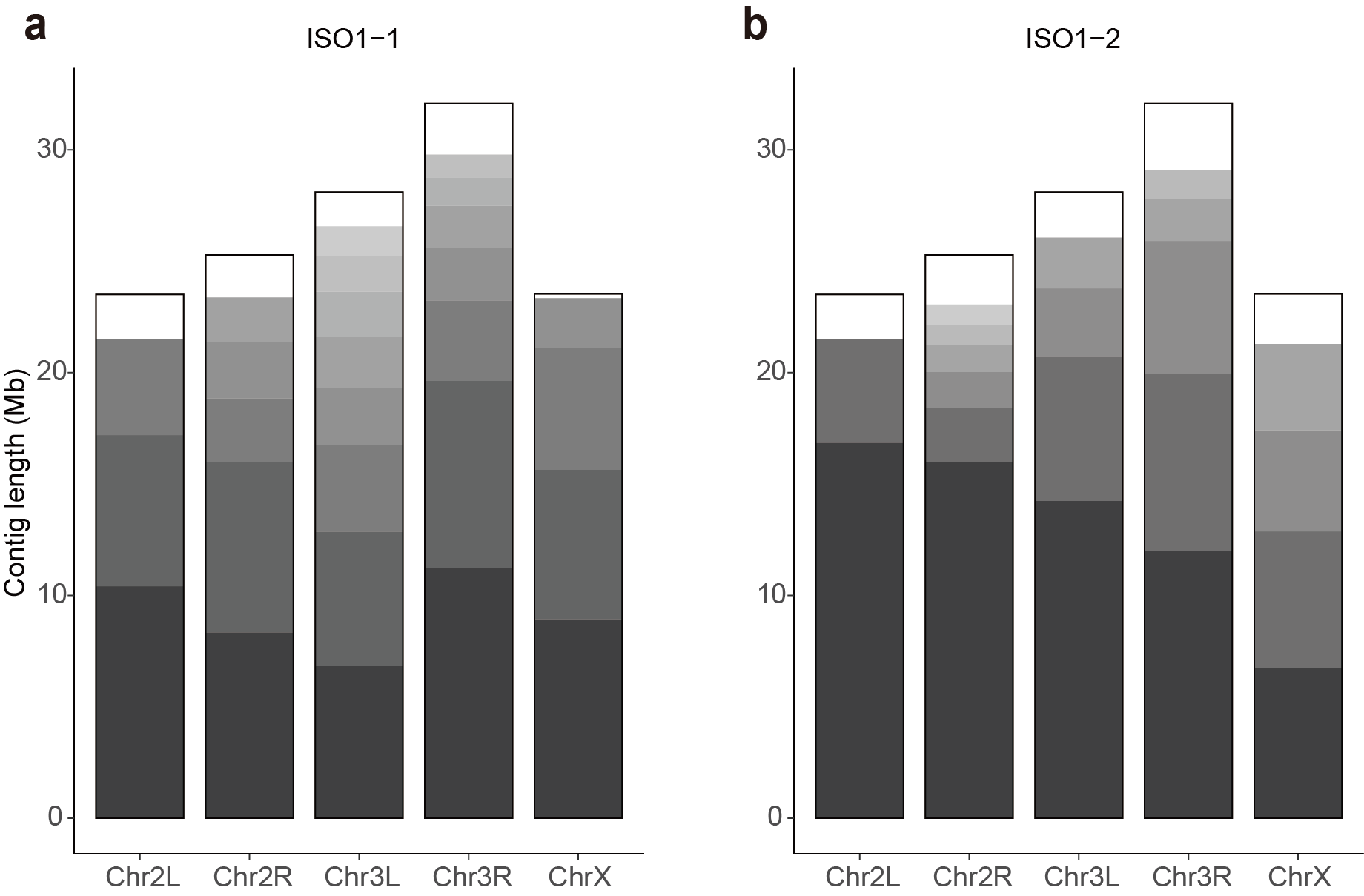


**Supplementary Fig. 5 | Number of contigs covering over 90% (LG90) of five main arms.**

Distribution of LG90 across main arms for ISO1-1 (**a**) and ISO1-2 (**b**). Grey bars with gradients denote contigs ordered by sizes, while white bars depict unassembled sequences in the reference genome.


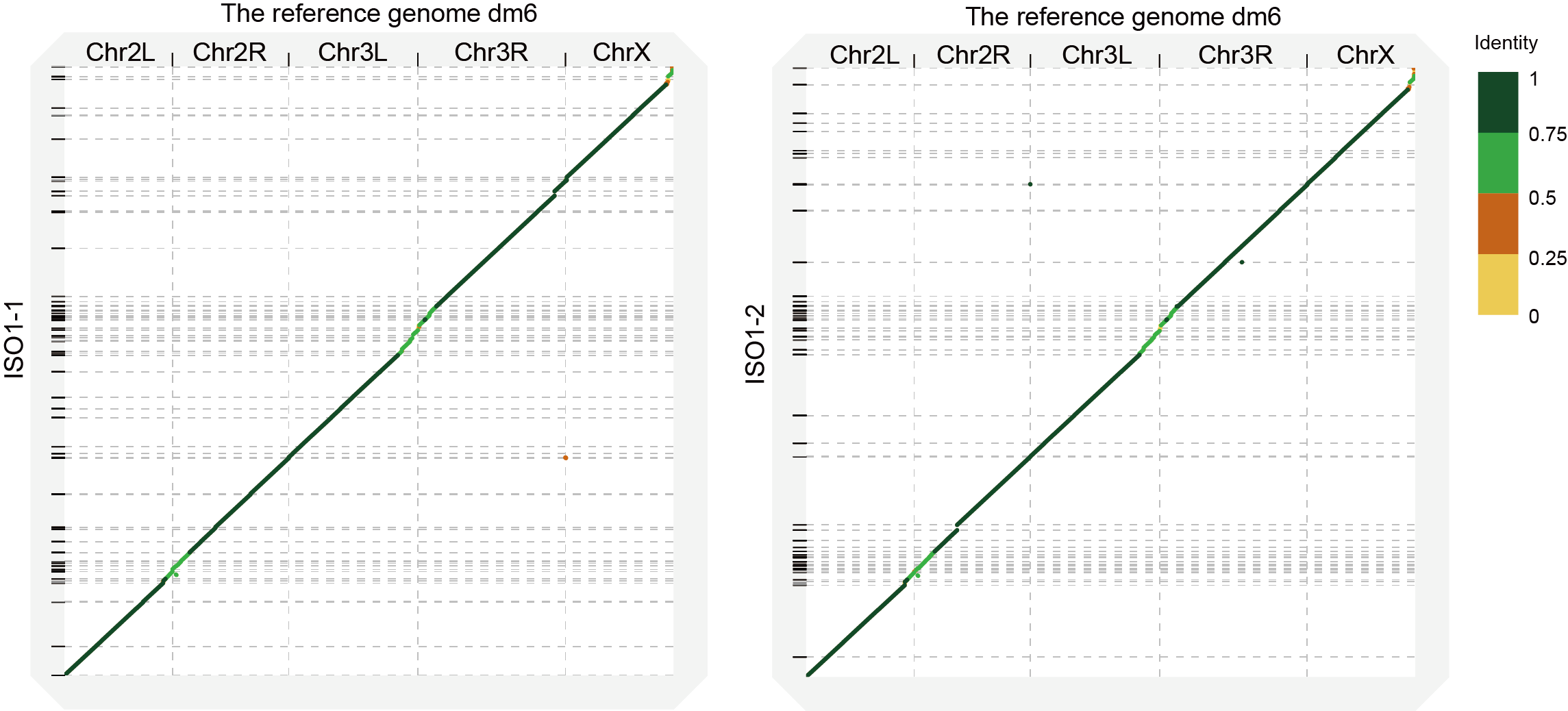


**Supplementary Fig. 6 | Dot plots between ISO1-1/2 contigs and the five main chromosome arms of the reference genome.**

Black lines in the left margin and corresponding dashed lines denote contig boundaries, while colors indicate four identity ranges. It is noteworthy that regions with low identity (*e.g.*, green) mapped by numerous short contigs predominantly correspond to repeat-rich centromeres (refer also to Fig. 2c).


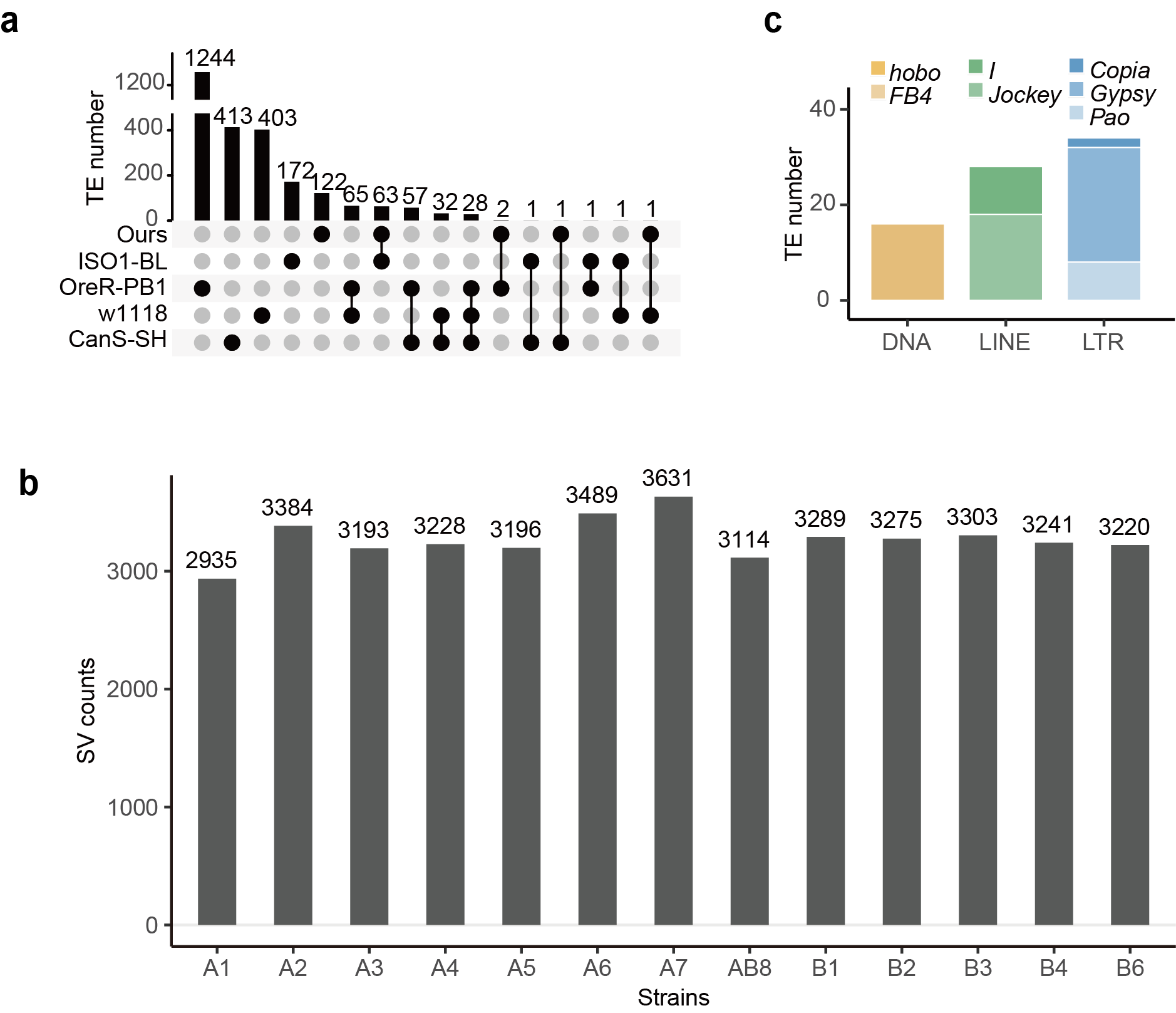


**Supplementary Fig. 7 | SVs in ISO1-1/2 and DSPR strains.**

**a,** An UpSet plot showing the distribution of TE insertions across different lab fly strains. The number of SVs is displayed above each bar. For example, 1,244 insertions (shown as a truncated bar) are specific to OreR-PB1, while 65 insertions are only present in OreR-PB1 and w1118. Note that ISO1-BL represents a recent isolate of the ISO1 strain obtained from the Bloomington *Drosophila* Stock Center. **b,** Counts of euchromatic SVs across 13 DSPR founder strains. The data were obtained from <https://github.com/mahulchak/dspr-asm/tree/master/variant_data>. The number of SVs is displayed above each bar. **c,** Distribution of transposon insertion types in the reference genome.


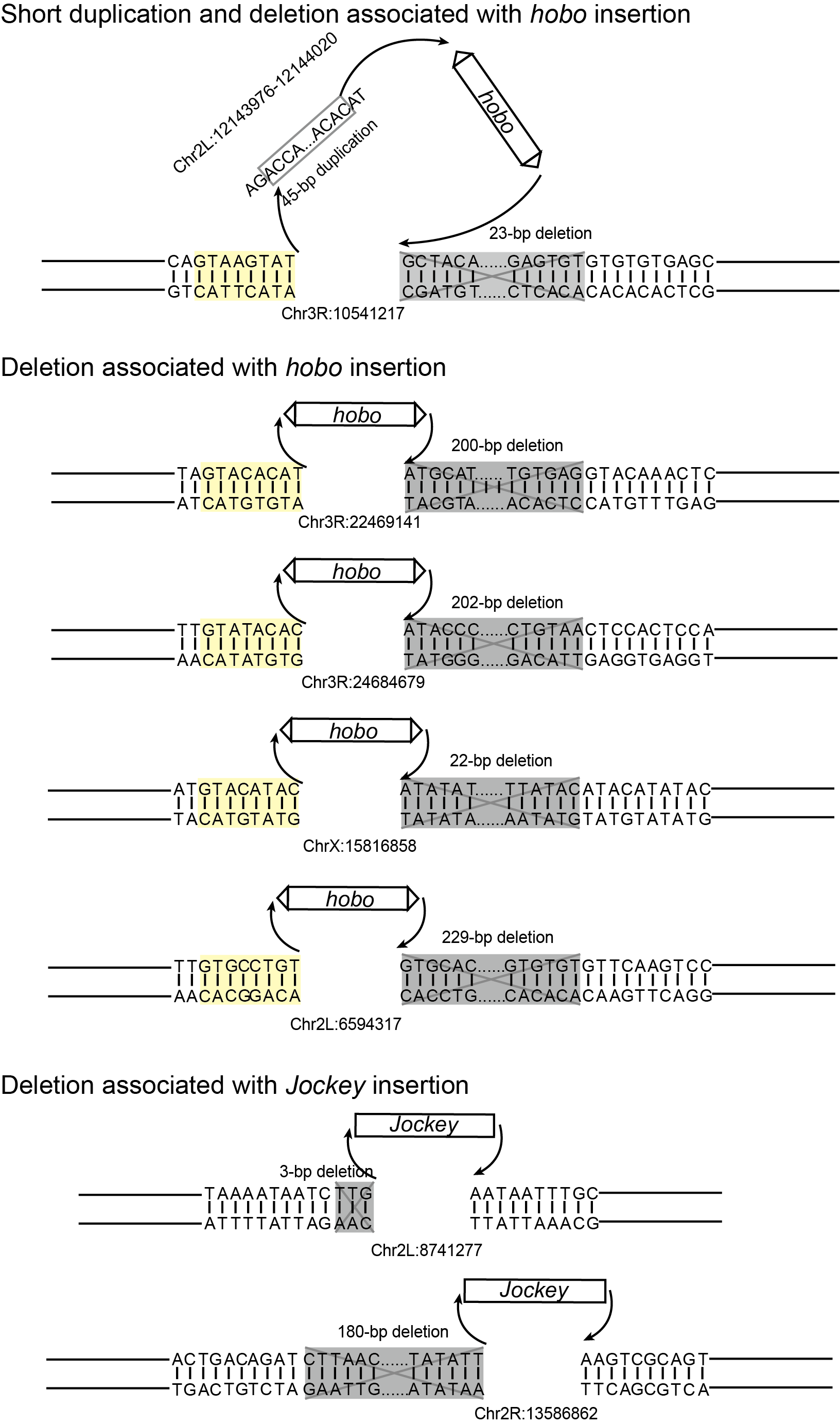


**Supplementary Fig. 8 | Complex transpositions with extra duplications and/or deletions.** The *hobo* binding motifs are marked in yellow, and deletions are marked in grey. In the top panel, the duplicated region is framed.


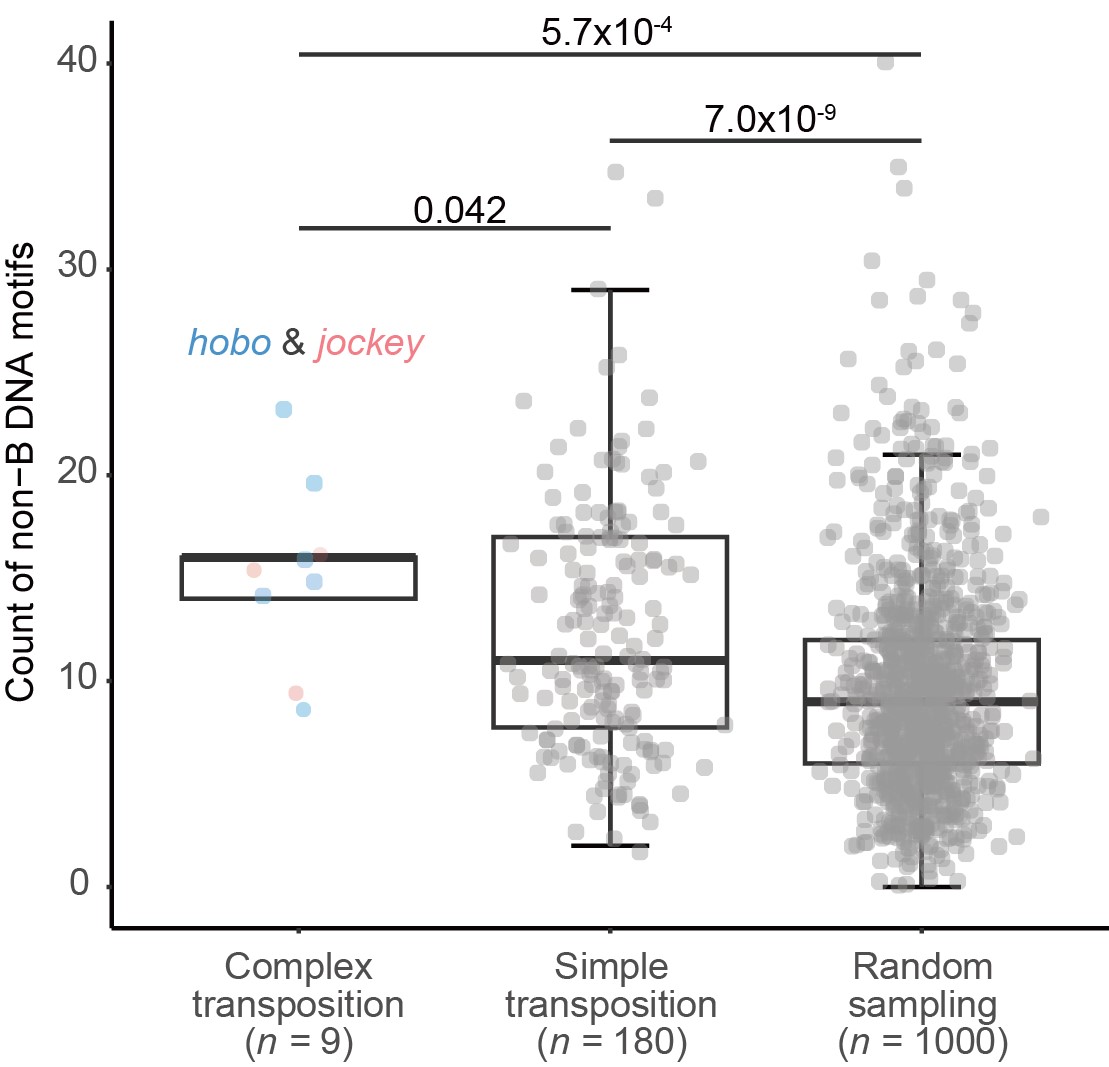


**Supplementary Fig. 9 | Distribution of non-B DNA motif counts at TE transposition sites (up- and downstream 1000-bp windows) and random locations.**

The figure conventions follow Fig. 3f.

**
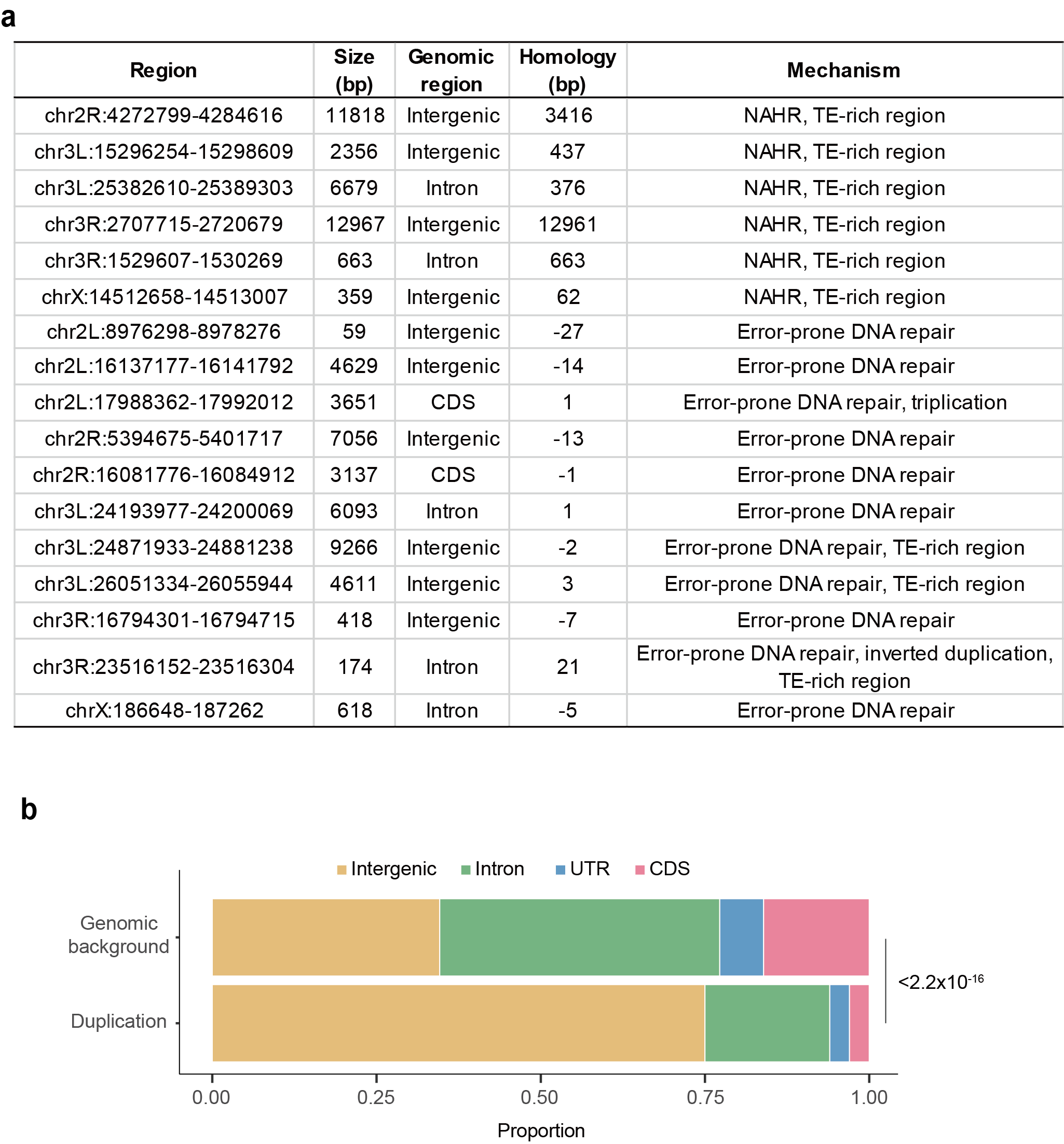
 Supplementary Fig. 10 | Duplications in ISO1-1/2.**

**a,** Summary of the 17 duplications mediated by different mechanisms. Negative values in the "Homology (bp)" column indicate *de novo* insertions at the middle breaking point of two duplication blocks. Homology sequences ranging from 1 to 30 bp were considered as microhomology^5^. "CDS" denotes coding sequence. **b,** Relative proportion of sequences across genomic regions. A proportional test was performed between duplication and genomic background, the *P* value was displayed.


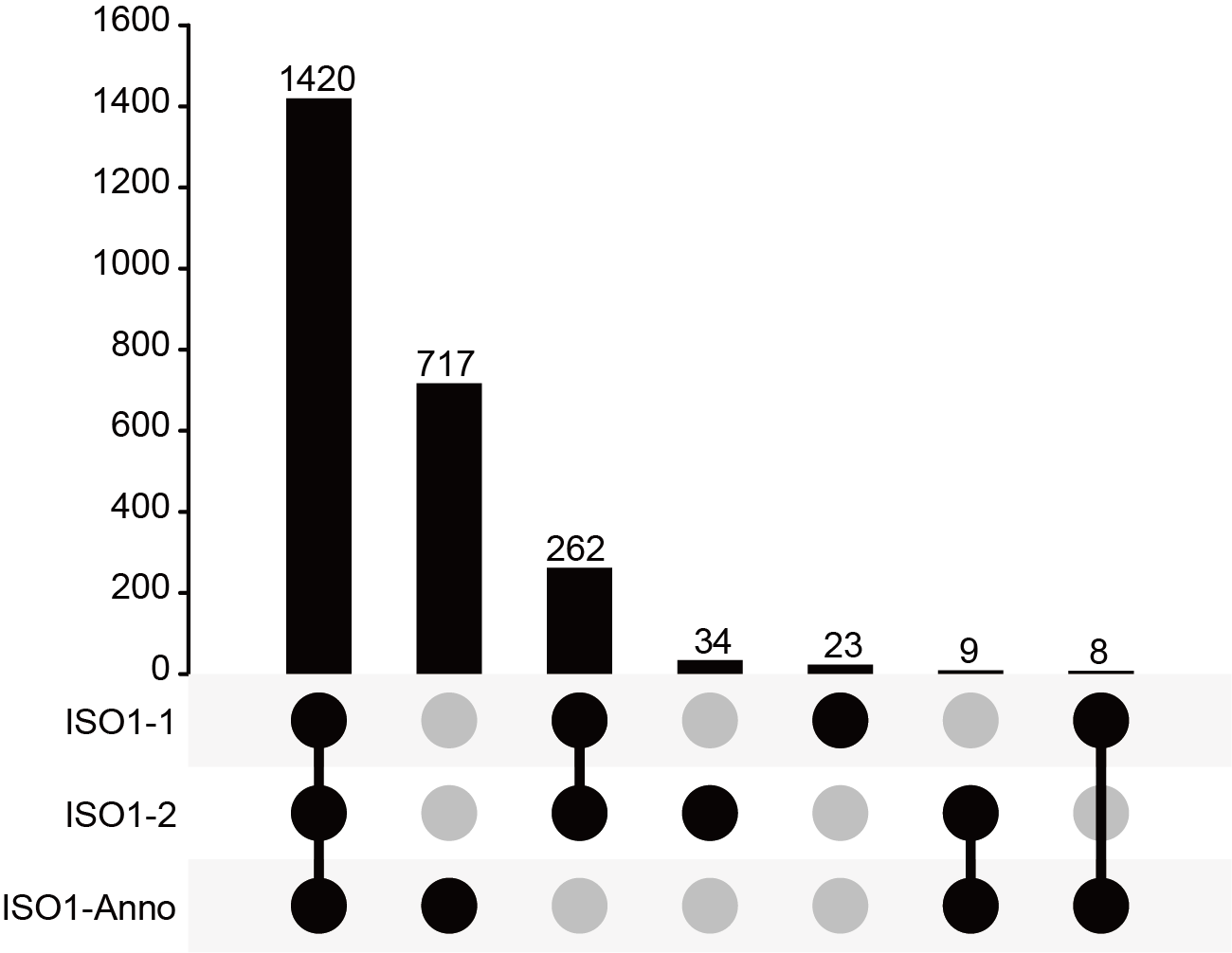


**Supplementary Fig. 11 | SNP distribution across different ISO1 individuals.**

The figure convention follows Supplementary Fig. 7a.


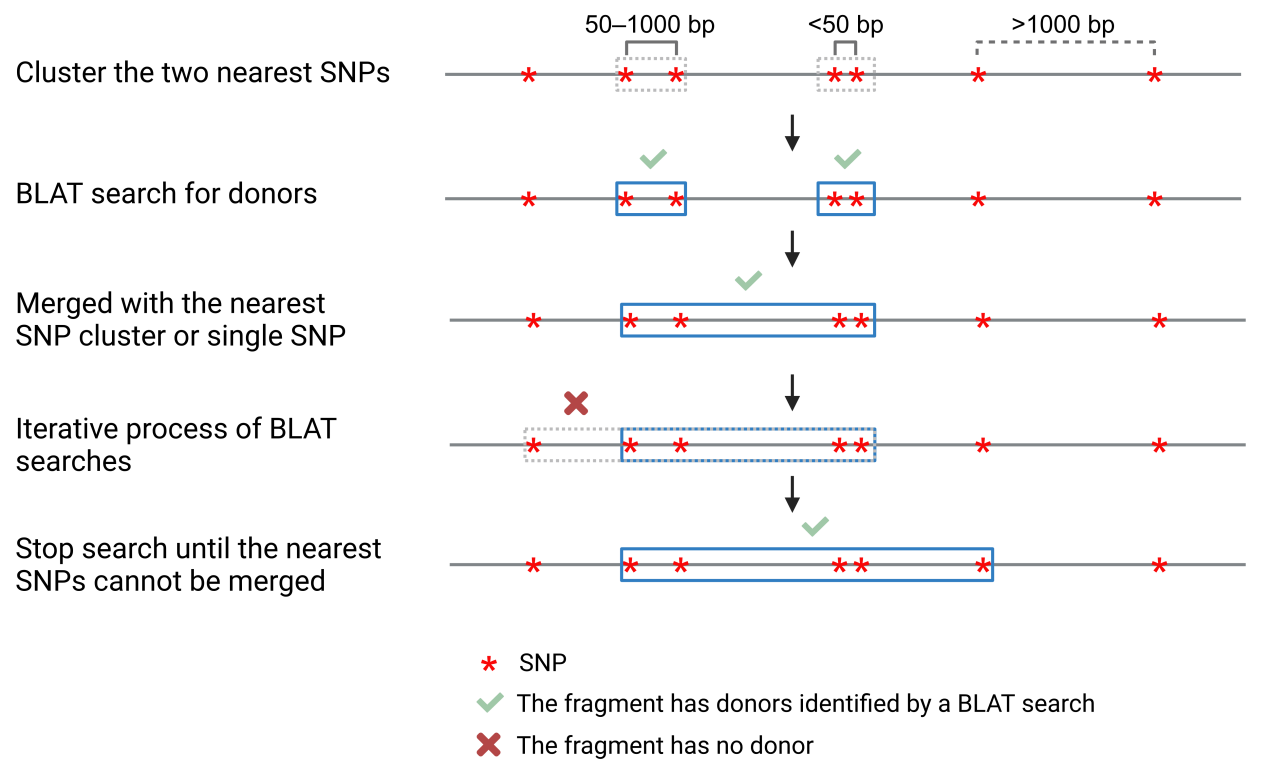


**Supplementary Fig. 12 | The pipeline for determining the boundaries of each conversion event.**

We developed an iterative pipeline to identify the boundaries of conversions. The first (leftmost) SNP could not be merged since no donor could be found. In contrast, the second to the sixth SNPs could be merged since one donor harboring all these SNPs could be identified.


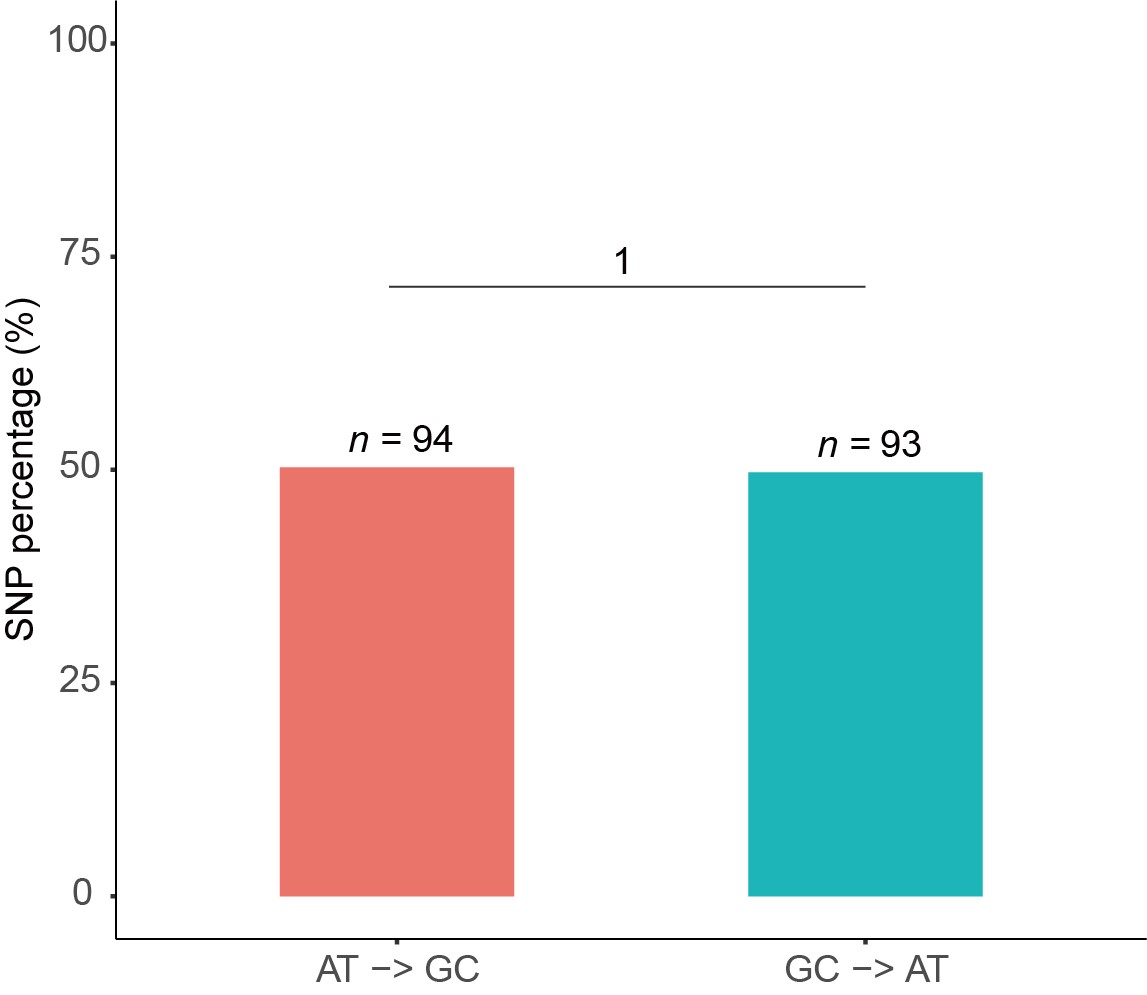


**Supplementary Fig. 13 | Absence of GC bias in TE conversions.**

The mutation direction of the SNPs at conversion acceptor sites is summarized as AT to GC and GC to AT. AT -> GC denotes the conversion from A or T to G or C, encompassing changes like A to G, A to C, T to G, and T to C. The same principles apply to GC -> AT. Statistical significance was evaluated using a two-sided binomial test.

**Supplementary References**

1. Fan X*, et al.* SMOOTH-seq: single-cell genome sequencing of human cells on a third-generation sequencing platform. *Genome Biol* **22**, 195 (2021).

2. Hard J*, et al.* Long-read whole-genome analysis of human single cells. *Nat Commun* **14**, 5164 (2023).

3. Kingan SB*, et al.* A high-quality de novo genome assembly from a single mosquito using PacBio sequencing. *Genes* **10**, 62 (2019).

4. Schneider C*, et al.* Two high-quality de novo genomes from single ethanol-preserved specimens of tiny metazoans (Collembola). *Gigascience* **10**, giab035 (2021).

5. Conrad DF*, et al.* Mutation spectrum revealed by breakpoint sequencing of human germline CNVs. *Nat Genet* **42**, 385-391 (2010).
