# Supplementary material for: Low-input PacBio sequencing generates high-quality individual fly genomes and characterizes mutational processes
Source: Nat Commun. 2024 Jul 5;15:5644. doi: 10.1038/s41467-024-49992-6 (PMC11226609; doi:10.1038/s41467-024-49992-6)
Supplement: Supplementary file 3 — Description of Supplementary Data Files [file 41467_2024_49992_MOESM3_ESM.docx]

**Description of Additional Supplementary Files**

**File Name: Supplementary Data 1**

**Description: Supplementary Data 1. The data yields of two multiplexed HiFi sequencing libraries.** The FASTA files containing CCS reads were used to calculate the data size for each sample. Barcode IDs were the same as those in Supplementary Data 2. SD stands for standard deviation.

**File Name: Supplementary Data 2**

**Description: Supplementary Data 2. The adapter sequences with unique barcodes for multiplexed libraries.** Hairpin adapters with unique barcodes were used in the multiplexed libraries. “Phos” indicates the phosphate group at the 5’ end of the DNA molecule. The pair of fragments in bold black represent barcode sequences and reverse complementary sequences. The 19 bp sequences at the two terminals represent Tn5 binding sites (mosaic end or ME) and their reverse complementary sequences. Sometimes, barcodes could overlap with ME by one or two nucleotides.

**File Name: Supplementary Data 3**

**Description: Supplementary Data 3. Evaluation of ISO1-1/2 assemblies with decreasing depth and aISO1-Anno/PB assemblies with increasing depth.** The table conventions follow Table 1.

**File Name: Supplementary Data 4**

**Description: Supplementary Data 4. Copy number and gene arrangement of *Sdic* locus among different assemblies of the ISO1 strain.** As a tandem gene family, *Sdic* is positioned on chrX, flanked by *AnxB10* and *sw*. In the ISO1 reference genomes, releases five (UCSC dm5) and six (UCSC dm6), this gene family is represented by four and seven copies, respectively. Conversely, all other four genome assemblies contain six copies in an identical order. In addition, in dm5 and dm6, the positions of *Sdic2* and *Sdic4* are exchanged compared to other assemblies. Notably, Berlin *et al.* (2015) and Kim *et al.* (2014) sequenced the same ISO1 subline used for the *Drosophila* Genome Project, which produced the reference genome. Despite generating the original sequencing data, they did not resolve the *Sdic* locus. However, Clifton *et al.* (2017) successfully resolved it in both projects.

**File Name: Supplementary Data 5**

**Description: Supplementary Data 5. SV validation in aISO1-Anno.** The table presents nine complex transpositions as well as 80 randomly selected ISO1-1/2 shared SVs. The "Description" column provides details of each SV. The "Simple/Complex" column distinguishes nine complex transpositions. The "State in aISO1-Anno" column indicates whether the SV has been confirmed present in the aISO1 annotation ("Present") or *vice versa* ("Absent").

**File Name: Supplementary Data 6**

**Description: Supplementary Data 6. Gene conversion events in TEs.** “TE type” indicates the name, family, and class of TE; “TE divergence (%)” indicates the divergence between the copy and the consensus TE sequence; and “TE length ratio (%)” is calculated by the length of the mappable TE fragment divided by the total length of the consensus TE sequence. “Count” indicates the number of candidate donors. A value equal to 1 indicates a conversion with a unique donor. For conversions with multiple donors, the donor with the minimal distance from the acceptor was chosen. For interarm or interchromosomal events, the donor was chosen randomly. The “Membership score” follows ref.^1^, indicating the expression level of TE in fly spermatocytes. “NA” means that the TE is not included in the previous analyses^1^.

**File Name: Supplementary Data 7**

**Description: Supplementary Data 7. Contigs without taxonomic annotation**. For contigs lacking taxonomic annotation, “Y-linked” indicates that the contig is inferred to be a chrY fragment (Materials and methods). The remaining ones represent VNTRs with their potential locations in the reference genome and the annotation of consensus units. It is noteworthy that three VNTRs could not be mapped back to the reference genome.

**Supplementary References**

1. Lawlor MA, Cao W, Ellison CE. A transposon expression burst accompanies the activation of Y-chromosome fertility genes during Drosophila spermatogenesis. *Nat Commun* **12**, 6854 (2021).
