# Supplementary material for: Low-input PacBio sequencing generates high-quality individual fly genomes and characterizes mutational processes
Source: Nat Commun. 2024 Jul 5;15:5644. doi: 10.1038/s41467-024-49992-6 (PMC11226609; doi:10.1038/s41467-024-49992-6)
Supplement: Supplementary file 11 — Reporting Summary [file 41467_2024_49992_MOESM11_ESM.pdf]

nature portfolio

Corresponding author(s): Hangxing Jia
Shengjun Tan
Jue Ruan
Yong E. Zhang

Last updated by author(s): May 27, 2024

Reporting Summary

Nature Portfolio wishes to improve the reproducibility of the work that we publish. This form provides structure for consistency and transparency in reporting. For further information on Nature Portfolio policies, see our Editorial Policies and the Editorial Policy Checklist.

Statistics

For all statistical analyses, confirm that the following items are present in the figure legend, table legend, main text, or Methods section.

n/a	Confirmed	
☐	☒	The exact sample size (*n*) for each experimental group/condition, given as a discrete number and unit of measurement
☐	☒	A statement on whether measurements were taken from distinct samples or whether the same sample was measured repeatedly
☐	☒	The statistical test(s) used AND whether they are one- or two-sided<br>*Only common tests should be described solely by name; describe more complex techniques in the Methods section.*
☒	☐	A description of all covariates tested
☒	☐	A description of any assumptions or corrections, such as tests of normality and adjustment for multiple comparisons
☐	☒	A full description of the statistical parameters including central tendency (e.g. means) or other basic estimates (e.g. regression coefficient) AND variation (e.g. standard deviation) or associated estimates of uncertainty (e.g. confidence intervals)
☐	☒	For null hypothesis testing, the test statistic (e.g. *F*, *t*, *r*) with confidence intervals, effect sizes, degrees of freedom and *P* value noted<br>*Give P values as exact values whenever suitable.*
☒	☐	For Bayesian analysis, information on the choice of priors and Markov chain Monte Carlo settings
☒	☐	For hierarchical and complex designs, identification of the appropriate level for tests and full reporting of outcomes
☒	☐	Estimates of effect sizes (e.g. Cohen's *d*, Pearson's *r*), indicating how they were calculated

Our web collection on statistics for biologists contains articles on many of the points above.

Software and code

Policy information about availability of computer code

Data collection	PacBio Sequel II HiFi was used for generating sequencing data.
Data analysis	Public softwares: Python v3.11.4; R v4.2.1; Minimap2 v2.24; BWA v0.7.17; CCS v6.4.0; Hifiasm v0.12; Racon v1.6.0; merfin v1.1; BCFtools v1.14; Winnowmap2 v2.03; SAMtools v1.17; Sniffiles2 v2.0.6; QUAST v5.2.0; BUSCO v5.4.2; Flye v2.9; Merqury v1.3; BLAST v2.12.0; BLAT v37x1; MUMmer v4.0.0; LASTZ v1.04.15; svmu; BEDTools v2.31.0; non-B-gfa; PAV v2.3.4; SeqKit v2.4.0; TBtools-II v1.120137; DIAMOND v2.0.13; IGV v2.13.2126; Blobtools2 v2.6.5; TRF v4.09; Snakemake v8.0.1.<br>Scripts developed for this study: GC_bias_2.pl; ccs_circle.count.final.py; downsampling.py; relative_depth_2.pl; rmdup.py; SV_results_filter.pl; ccs_identity.final.py; non-B_DNA_sum2.py; relative_depth_3.pl; select_XY.median.py; sv.C01.ccs.hifiasm.txt; GC_bias_1.pl; Tn5_insertion_site_bias.pl; relative_depth_1.pl; shared_sp.final.py; combine_mnp_files.same_reads.py; filter_sa_bam1.py; out_overlap_bed.py; validated_non_rmsk_satellite_simple_trf_snp.baseq0.mapq0.py; filter_sa_bam.py; get_assembly_snp_vaf.baseq0.mapq0.py; same_reads_phasing.py.<br>All scripts developed for this study are available at https://github.com/Zhanglab-IOZ/LILAP and archived in Zenodo.

For manuscripts utilizing custom algorithms or software that are central to the research but not yet described in published literature, software must be made available to editors and reviewers. We strongly encourage code deposition in a community repository (e.g. GitHub). See the Nature Portfolio guidelines for submitting code & software for further information.

Data

Policy information about availability of data

All manuscripts must include a data availability statement. This statement should provide the following information, where applicable:

- Accession codes, unique identifiers, or web links for publicly available datasets
- A description of any restrictions on data availability
- For clinical datasets or third party data, please ensure that the statement adheres to our policy

The LILAP and amplification-based aISO1-Anno sequencing data, along with the final assemblies generated in this study, have been concurrently deposited in the NCBI Bioproject database under accession code PRJNA983717 [https://www.ncbi.nlm.nih.gov/bioproject/?term=PRJNA983717] and in the National Genomics Data Center (part of the China National Center for Bioinformation) under accession code PRJCA019897 [https://ngdc.cncb.ac.cn/bioproject/browse/ PRJCA019897]. Three public datasets used in this study are available in the NCBI Sequence Read Archive (SRA): amplification-based CCS data from the PacBio company under accession code SRR12473480 [https://www.ncbi.nlm.nih.gov/sra/?term=SRR12473480], Tn5 tagmentation-based short-read data under accession code SRX7201057 [https://www.ncbi.nlm.nih.gov/sra/?term=SRX7201057], and conventional PacBio bulk HiFi sequencing data under accession code SRR10238607 [https://www.ncbi.nlm.nih.gov/sra/?term=SRR10238607].

Research involving human participants, their data, or biological material

Policy information about studies with human participants or human data. See also policy information about sex, gender (identity/presentation), and sexual orientation and race, ethnicity and racism.

Reporting on sex and gender	Not involving human materials.
Reporting on race, ethnicity, or other socially relevant groupings	Not applicable
Population characteristics	Not applicable
Recruitment	Not applicable
Ethics oversight	Not applicable

Note that full information on the approval of the study protocol must also be provided in the manuscript.

Field-specific reporting

Please select the one below that is the best fit for your research. If you are not sure, read the appropriate sections before making your selection.

☒ Life sciences ☐ Behavioural & social sciences ☐ Ecological, evolutionary & environmental sciences

For a reference copy of the document with all sections, see nature.com/documents/nr-reporting-summary-flat.pdf

Life sciences study design

All studies must disclose on these points even when the disclosure is negative.

Sample size	Sample sizes were determined empirically: 1) during random downsampling, 15x depth was chosen. 2) during non-B DNA analyses, 1000 random regions with 400-bp or 2000-bp lengths were sampled; 3) to confirm that the individual-specific SVs tend to be absent in the fly families data, 20 random SVs were manually examined; and 4) to estimate the expectation of count of cSNPs in the genome, SNPs with the same number of cSNPs were randomly simulated in the genome for 100,000 times.
Data exclusions	In Fig. 2b, outliers were removed to simplify the plot. Specifically, any data point situated more than 1.5 times the interquartile range (IQR) either above the third quartile (Q3) or below the first quartile (Q1) was classified as an outlier.
Replication	Two single male replicates were used for genome sequencing and assembling. Two fly family replicates, each with both parents and four offspring including two males and two females, were used for genome sequencing. Five technical replicates were used in Supplementary Fig. 2a, and six single-fly biological replicates were used in Supplementary Fig. 2d.
Randomization	Randomization was implemented in five aspects. Firstly, during downsampling-based genome assembly, 15x random sequencing data of two LILAP-based flies and two amplification-based flies were selected, and 60x random sequencing data of two amplification-based flies were selected. Secondly, for non-B DNA motif analyses, we randomly chose 1000 fragments with lengths of 400 bp and 2000 bp as the random background. Thirdly, to verify that individual-specific SVs tend to be absent in the fly families data, 20 random SVs shared by two individual flies were sampled for manual heterozygosity assessment. Fourthly, to evaluate the random occurrence of cSNPs in the genome, SNPs with an equal number of cSNPs in each bin were randomly chosen 100,000 times. Lastly, to evaluate SV calling quality, 80 randomly selected ISO1-1/2 shared SVs were manually examined in the aISO1-Anno amplification data.

Blinding | Blinding was largely irrelevant to our study. Both data collection and analyses needed an understanding of the nature of the samples.

Reporting for specific materials, systems and methods

We require information from authors about some types of materials, experimental systems and methods used in many studies. Here, indicate whether each material, system or method listed is relevant to your study. If you are not sure if a list item applies to your research, read the appropriate section before selecting a response.

Materials & experimental systems

n/a	Involved in the study
☒	☐ Antibodies
☒	☐ Eukaryotic cell lines
☒	☐ Palaeontology and archaeology
☐	☒ Animals and other organisms
☒	☐ Clinical data
☒	☐ Dual use research of concern
☒	☐ Plants

Methods

n/a	Involved in the study
☒	☐ ChIP-seq
☒	☐ Flow cytometry
☒	☐ MRI-based neuroimaging

Animals and other research organisms

Policy information about studies involving animals; ARRIVE guidelines recommended for reporting animal research, and Sex and Gender in Research

Laboratory animals	Drosophila melanogaster ISO1 reference strain was maintained on standard food in incubators with conditions specified at 25°C, 60% relative humidity and 12h:12h light/dark cycle. We collected single adult male flies and fly families (the parents and four offspring) to extract genome DNA.
Wild animals	The study did not involve wild animals.
Reporting on sex	Three single males from D. melanogaster ISO1 reference strain were selected for genome sequencing and assembling. Two fly families, each with both parents and four offspring including two males and two females, were used for genome sequencing. Sequencing depth of female and male family sequencing data on all contigs was used to determine Y-linked contigs.
Field-collected samples	The study did not involve samples collected from the field.
Ethics oversight	No ethical approval or guidance was required since we did not perform relevant experiment.

Note that full information on the approval of the study protocol must also be provided in the manuscript.

Plants

Seed stocks	The study did not involve plant samples.
Novel plant genotypes	The study did not involve plant samples.
Authentication	The study did not involve plant samples.
