# Supplementary figures and images for: Low-input PacBio sequencing generates high-quality individual fly genomes and characterizes mutational processes
Source: Nat Commun. 2024 Jul 5;15:5644. doi: 10.1038/s41467-024-49992-6 (PMC11226609; doi:10.1038/s41467-024-49992-6)

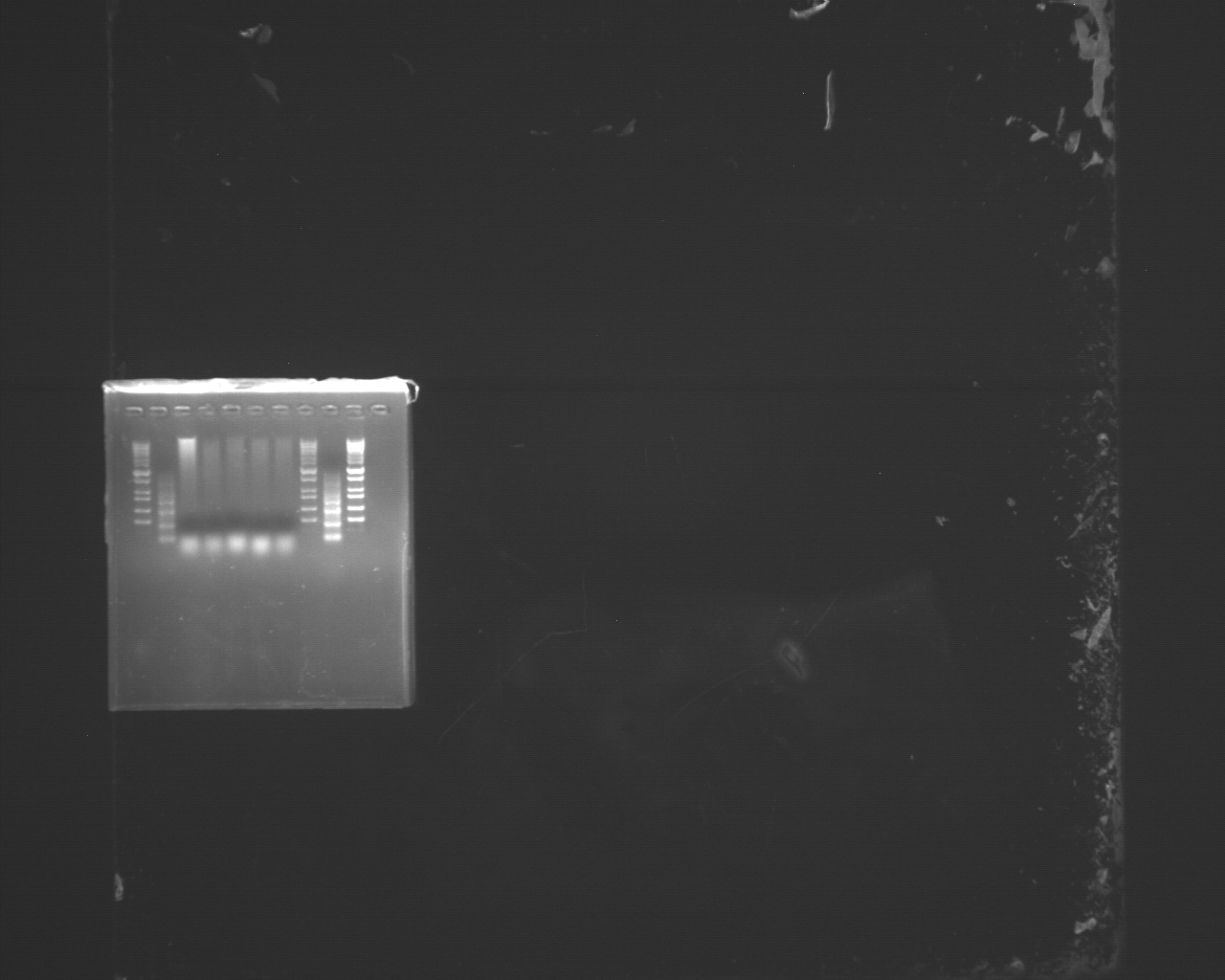

Supplement: Supplementary file 12 — Source Data [file 41467_2024_49992_MOESM12_ESM.zip › Supplementary Fig 2a.Tif]

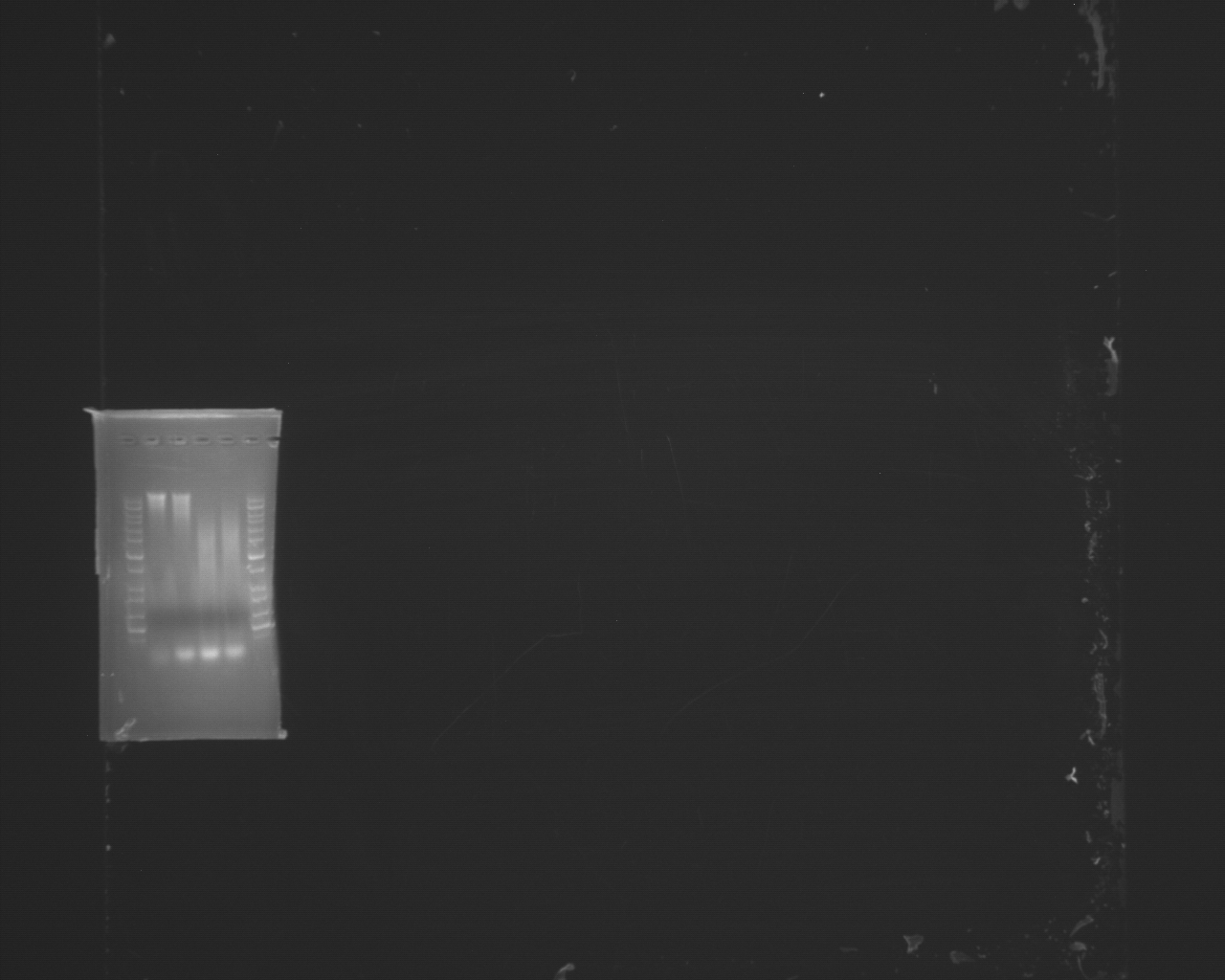

Supplement: Supplementary file 12 — Source Data [file 41467_2024_49992_MOESM12_ESM.zip › Supplementary Fig 2b.Tif]

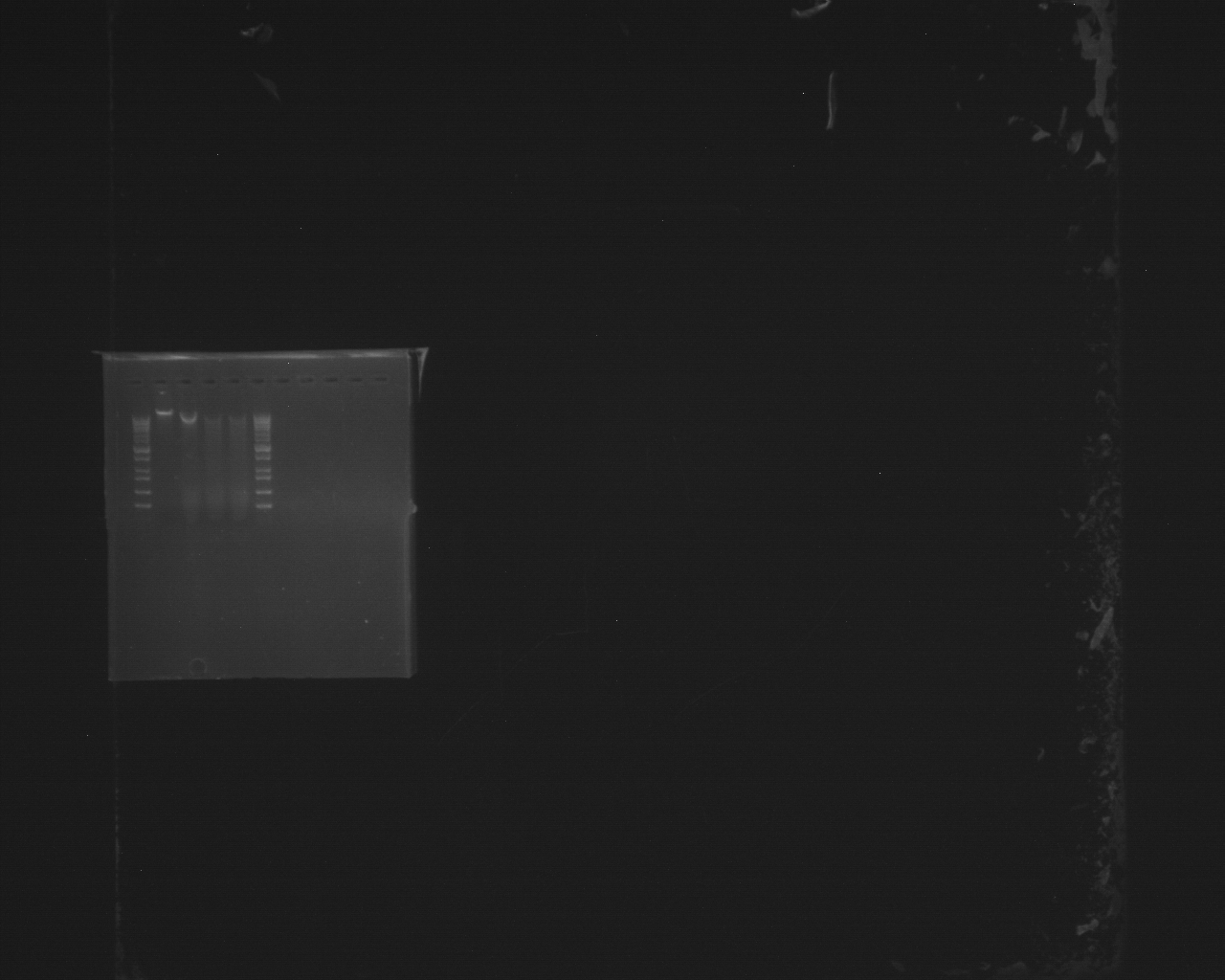

Supplement: Supplementary file 12 — Source Data [file 41467_2024_49992_MOESM12_ESM.zip › Supplementary Fig 2c.Tif]

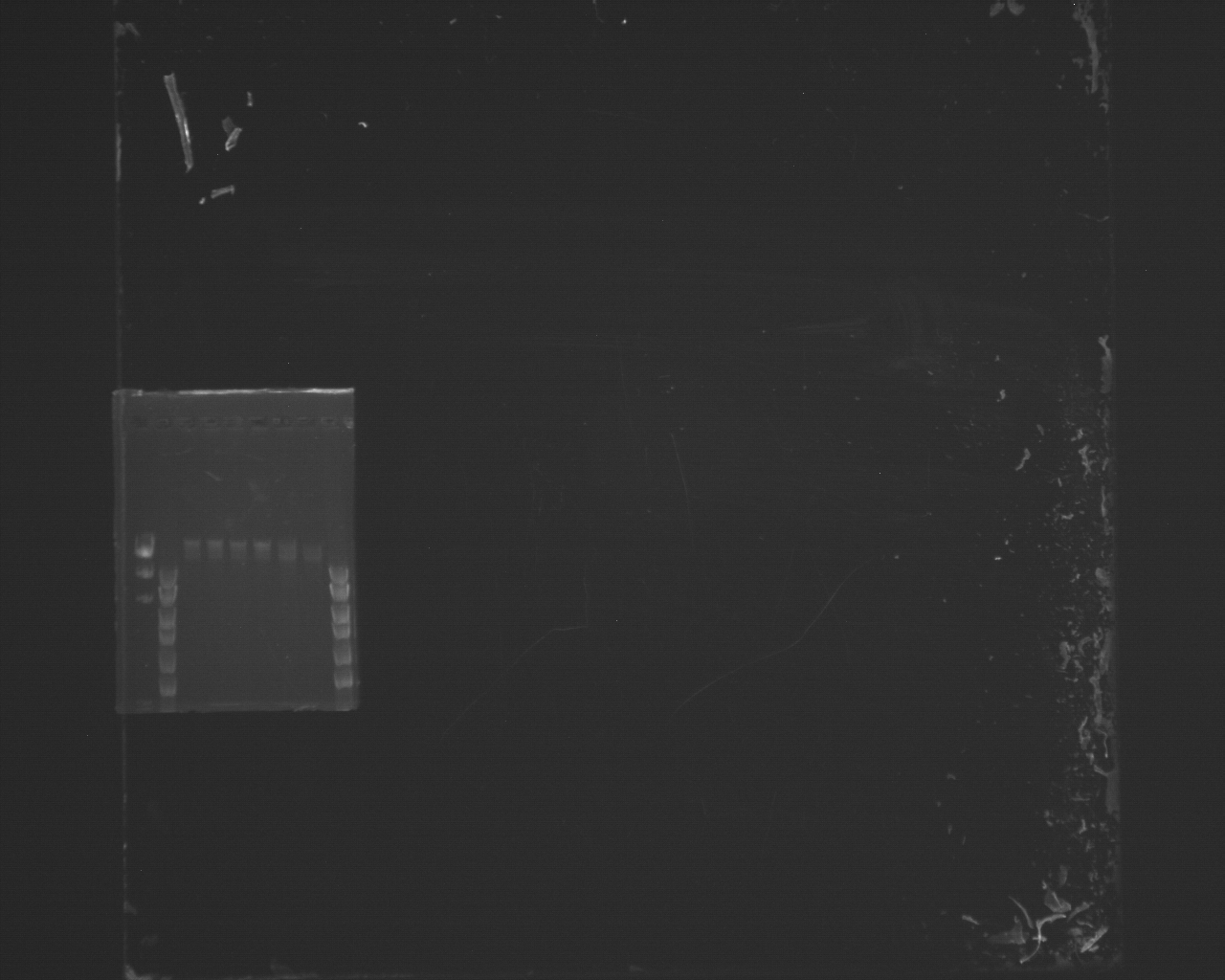

Supplement: Supplementary file 12 — Source Data [file 41467_2024_49992_MOESM12_ESM.zip › Supplementary Fig 2d.tif]
